# Acceptance of COVID-19 vaccination during pregnancy among Thai pregnant women and their spouses: a prospective survey

**DOI:** 10.1186/s12978-022-01383-0

**Published:** 2022-03-24

**Authors:** Kotchakorn Pairat, Chadakarn Phaloprakarn

**Affiliations:** 1 Department of Nursing, Faculty of Medicine Vajira Hospital, Navamindradhiraj University, Bangkok, Thailand; 2 Department of Obstetrics and Gynecology, Faculty of Medicine Vajira Hospital, Navamindradhiraj University, 681 Samsen Road, Dusit, Bangkok, 10300 Thailand

**Keywords:** Coronavirus disease 2019, COVID-19 vaccine, Pregnancy, Pregnant women, SARS-CoV-2, Spouses, Vaccine acceptance

## Abstract

**Background:**

Vaccination is one of the most reliable interventions against coronavirus disease 2019 (COVID-19). Although pregnant women’s attitudes toward COVID-19 vaccination are well studied, husbands’ views toward COVID-19 vaccination for these women have not been surveyed. We aimed to determine the rates and associated factors of accepting attitudes toward COVID-19 vaccination during pregnancy among Thai pregnant women and their spouses and to evaluate the actual rate of vaccination during pregnancy among these women.

**Methods:**

A prospective survey was conducted at a tertiary care center in Bangkok, Thailand. A total of 176 Thai pregnant woman/husband dyads who attended our antenatal care clinic between 1 July 2021 and 30 September 2021 were consecutively recruited for evaluations of their accepting attitudes toward COVID-19 vaccination during pregnancy. After delivery, data on COVID-19 vaccination during pregnancy among pregnant women were extracted from the hospital’s electronic database.

**Results:**

After exclusion of five pregnant women with prepregnancy COVID-19 vaccination, 171 women and 176 male partners were included. The rates of accepting attitudes toward COVID-19 vaccination during pregnancy were 60.8% and 61.4%, respectively. Multivariate analysis showed that having a husband who favored COVID-19 vaccination for his wife was independently associated with COVID-19 vaccine acceptance among pregnant women (adjusted odds ratio 4.82; 95% confidence interval 2.34, 9.94). However, confidence in vaccine safety was an associated factor of the husband’s willingness to have his wife vaccinated for COVID-19 during pregnancy (adjusted odds ratio 12.56; 95% confidence interval 2.35, 67.18). The actual rate of vaccination during pregnancy was 88.3%.

**Conclusions:**

Although the rates of accepting attitudes toward COVID-19 vaccination during pregnancy among Thai pregnant women and their spouses were modest, the actual rate of being vaccinated during pregnancy was high.

## Plain language summary

Vaccination is an important tool to fight against the coronavirus disease 2019 (COVID-19) pandemic. In this study, we investigated the rates and associated factors of accepting attitudes toward COVID-19 vaccination during pregnancy among Thai pregnant women and their spouses. The actual rate of vaccination during pregnancy among the enrolled women was also evaluated. We recruited 176 Thai pregnant woman/husband dyads who attended our antenatal care clinic between 1 July 2021 and 30 September 2021. The results showed that 60.8% of pregnant women and 61.4% of husbands had accepting attitudes toward COVID-19 vaccination during pregnancy. Having a husband who favored COVID-19 vaccination for his wife was a significant factor for COVID-19 vaccine acceptance among pregnant women, whereas confidence in vaccine safety was a significant predictor of the husband’s willingness to have his wife vaccinated for COVID-19 during pregnancy. The majority (96.2%) of the women who accepted vaccination were vaccinated against COVID-19 during pregnancy. On the other hand, 76.1% of the women who were initially reluctant to be vaccinated ultimately received COVID-19 vaccines during pregnancy. The actual rate of being vaccinated among the enrolled women was 88.3%. Given the results of this study, physicians, hospital administrators, and policymakers should focus on those who show vaccine hesitancy or refusal and implement intensive interventions because there is a possibility to change their attitudes if they have more knowledge and gain more trust in the vaccines.

## Introduction

The outbreak of coronavirus disease 2019 (COVID-19), caused by severe acute respiratory syndrome coronavirus 2 (SARS-CoV-2), has created an unprecedented global crisis with devastating health and socioeconomic impacts in every nation [1–3]. To fight against the COVID-19 pandemic, it is necessary to achieve herd immunity by vaccinating the global population as quickly as possible before the emergence and spread of new variants that can overcome immunity conferred by vaccines. Because COVID-19 vaccines have been developed and authorized for emergency use [4], public trust is a key element to the success of global vaccination.

Thailand is an upper middle-income Southeast Asian country that started a COVID-19 vaccination program with the CoronaVac and ChAdOx1 nCoV-19 vaccines in February 2021. Due to the limited number of vaccines available, the country has begun its vaccine rollout to medical personnel and frontline health workers, followed by persons at risk for severe COVID-19 illness, including those with chronic medical diseases as well as obese and elderly people. Aside from CoronaVac and ChAdOx1 nCoV-19, two different COVID-19 vaccines, involving BBIBP-CorV and BNT162b2, were started in June 2021 and August 2021, respectively, to accelerate vaccine uptake among vulnerable people and the general population.

In mid-2021, the Thai government launched a public relations campaign to encourage vaccination among pregnant women. This was due to a significant upsurge in the highly contagious Delta variant in the country, causing a rapid increase in new cases and a spike in deaths among vulnerable people, including pregnant persons. Healthcare providers were urged to emphasize discussions with pregnant women about the health risks associated with COVID-19 infection and the benefits of vaccination. At our institution, all pregnant women who attended the antenatal care (ANC) clinic would be advised or educated about the benefits of vaccination against COVID-19 [5–7]. ChAdOx1 nCoV-19 was the main vaccine provided to pregnant women in our hospital (and in the country) during that time. Although the vaccine was offered free of charge and only a few significant adverse events after vaccination were reported among the general population in Thailand, pregnant women might feel that it was too new and be concerned about its safety.

Many researchers have investigated the rates of accepting attitudes toward COVID-19 vaccination among pregnant people [8–13]. However, no one has explored the actual rate of being vaccinated during pregnancy among the women studied. In addition, husbands’ views about COVID-19 vaccination of their pregnant wives have not yet been surveyed. Since husbands, especially those in low- and middle-income Asian countries, are commonly involved in the decision-making of their wives, particularly on important matters [14–16], the husband’s acceptance or hesitation toward the COVID-19 vaccines offered to the wife might affect vaccine acceptance by the pregnant spouse.

The primary objective of this study was to determine the rates of accepting attitudes toward COVID-19 vaccination during pregnancy among Thai pregnant women and their spouses. The secondary objective was to identify the factors associated with the couples’ acceptance of COVID-19 vaccines and the actual rate of vaccination during pregnancy among the enrolled women.

## Methods

### Study design, setting and participants

This prospective survey-based study was conducted at the Faculty of Medicine Vajira Hospital. The hospital is an 800-bed tertiary care center located in Bangkok, Thailand, that facilitates approximately 2000 deliveries annually. The study was approved by the Vajira Institutional Review Board (certificate of approval no. 101/2564) and was performed in compliance with the Declaration of Helsinki.

The study involved pregnant women and their spouses who attended our ANC clinic between 1 July 2021 and 30 September 2021. The inclusion criteria for pregnant women and their husbands were Thai nationality, age at or above 18 years, and willingness to participate in the study. The exclusion criteria were pregnant women who (1) attended the clinic alone; (2) did not live with a partner; or (3) had already received at least one COVID-19 vaccine dose before the survey. Additionally, pregnant women or husbands who had experienced COVID-19 infection before enrollment were excluded from the study.

The sample size was calculated based on the findings of a prior survey, namely, a 60% rate of COVID-19 vaccine acceptance [13]. With a margin of error of 10%, a confidence level of 95%, and a 30% exclusion rate, at least 121 pregnant woman/husband dyads were needed for the study. To improve the reliability of research outcomes, we planned to enroll all couples attending our ANC clinic during the period from July to September 2021 who met the inclusion criteria. Hence, 176 couples were recruited.

### Procedure and study questionnaire

Consecutive pregnant women and their spouses who attended the ANC clinic during the study period and met the inclusion criteria were approached by a researcher. The aims of the study and the study procedure were explained before written informed consent was obtained. The wife and husband independently completed a written self-answered questionnaire in a separate space in the room with no interaction with the other. Assistance from a research assistant was available for any questions.

The questionnaire for all participants (pregnant women and their husbands) consisted of three parts. The first section contained basic sociodemographic and clinical data, including age, prepregnancy body mass index, gestational age, number of ANC visits, number of children, education, occupation, monthly income, public health insurance coverage, underlying diseases (diabetes/gestational diabetes, hypertension, obesity, cancer, and chronic lung, kidney, cardiovascular and cerebrovascular diseases), history of receiving a seasonal flu vaccine this year, history of receiving COVID-19 vaccine (only for husbands), presence or absence of relative(s) who had already been vaccinated against COVID-19, and presence or absence of relative(s) with COVID-19 infection.

In the second part, the participants responded to a question regarding levels of worry about COVID-19 infection by choosing 1 of 4 options [9]: ‘very worried’, ‘somewhat worried’, ‘not very worried’, and ‘not worried at all’. Both responses ‘very worried’ and ‘somewhat worried’ were combined into one category as ‘worry’, and both responses ‘not very worried’ and ‘not worried at all’ were combined into one category as ‘no worry’.

The third part comprised questions about COVID-19 vaccine acceptance, which included the most accepted vaccines, confidence in vaccination during pregnancy in terms of safety and efficacy, acceptance of vaccination during pregnancy, and the gestational age at which a respondent accepted vaccination. For questions about vaccine confidence and acceptance, responses were recorded on a 5-point Likert scale (‘strongly agree’, ‘agree’, ‘neutral’, ‘disagree’, and ‘strongly disagree’). Vaccine confidence and acceptance were defined as ‘yes’ if the responses were ‘strongly agree’ or ‘agree’ and as ‘no’ for any other responses.

During subsequent ANC visits until delivery, the women were asked whether they received COVID-19 vaccines. Data on their COVID-19 vaccination status during pregnancy were recorded in both paper and hospital electronic medical records.

### Outcome measures

The primary study outcome was an accepting attitude toward COVID-19 vaccination during pregnancy. The secondary outcomes were factors associated with COVID-19 vaccine acceptance and receiving COVID-19 vaccines during pregnancy.

### Statistical analysis

Data were analyzed using IBM SPSS Statistics for Windows, Version 22.0 (IBM Corporation, Armonk, NY, USA). Categorical data are described as numbers and percentages and were compared by means of the chi-square test or Fisher’s exact test, as appropriate. Continuous data are presented as the median and interquartile range (IQR) due to their nonnormal distributions and were compared by the Mann–Whitney U test. Variables that were significantly associated with COVID-19 vaccine acceptance in a univariate analysis were entered into a multivariate logistic regression analysis. The adjusted odds ratio (OR) and its 95% confidence interval (CI) were used to describe the odds of COVID-19 vaccine acceptance for each significant variable. The threshold for statistical significance was set at a 2-sided p < 0.05.

## Results

### Participant characteristics

A total of 176 couples (pregnant woman and husband) participated in this study during the study period. Of these, five women were excluded due to being vaccinated for COVID-19 before pregnancy. Hence, 171 women and 176 male partners were included in the analysis.

The median age of the 171 women included was 28 years (IQR 23–33 years), and the median gestational age when responding to the questionnaire was 26 weeks (IQR 18–31 weeks). Approximately half (54.4%) had at least one child. Three-quarters of them had no underlying disease, and 22.8% received a seasonal flu vaccine this year.

The median age of the spouses was 30 years (IQR 25–35 years). Most of them (92.6%) had no underlying disease, and 44.9% had already been vaccinated against COVID-19. The details of the participants’ characteristics are shown in Table 1.

**Table 1:** Baseline characteristics of pregnant women and their spouses

Characteristic	Pregnant women	Spouses
	(n = 171)	(n = 176)
Age (years)	28 (23–33)	30 (25–35)
Prepregnancy body mass index (kg/m^2^)		
< 18.5	25 (14.6)	–
18.5–24.9	82 (48.0)	–
25.0–29.9	37 (21.6)	–
≥ 30.0	27 (15.8)	–
Gestational age (weeks)	26 (18–31)	–
Number of ANC visits		
1–3	51 (29.8)	–
4–7	88 (51.5)	–
≥ 8	32 (18.7)	–
Number of children		
0	78 (45.6)	83 (47.1)
1	67 (39.2)	67 (38.1)
≥ 2	26 (15.2)	26 (14.8)
Education		
Primary education	14 (8.2)	13 (7.4)
Secondary education	97 (56.7)	105 (59.7)
College	16 (9.4)	17 (9.6)
Bachelor’s degree or higher	44 (25.7)	41 (23.3)
Occupation		
Public officer	18 (10.5)	32 (18.2)
Business owner	17 (9.9)	22 (12.5)
Employee	76 (44.5)	116 (65.9)
Unemployment	60 (35.1)	6 (3.4)
Monthly income		
< $500	106 (62.0)	65 (36.9)
$500–$999	58 (33.9)	94 (53.4)
≥ $1000	7 (4.1)	17 (9.7)
Public health insurance coverage		
Yes	158 (92.4)	64 (36.4)
Civil servant medical benefit scheme	18 (10.5)	26 (14.8)
Social security scheme	15 (8.8)	14 (8.0)
Universal coverage scheme	125 (73.1)	24 (13.6)
No	13 (7.6)	112 (63.6)
Underlying disease^a^		
Diabetes/gestational diabetes	19 (11.1)	3 (1.7)
Hypertension	2 (1.2)	5 (2.8)
Obesity	27 (15.8)	5 (2.8)
Other chronic diseases^b^	7 (4.1)	1 (0.6)
Any underlying disease		
Yes	43 (25.1)	13 (7.4)
No	128 (74.9)	163 (92.6)
History of receiving a seasonal flu this year		
Yes	39 (22.8)	16 (9.1)
No	132 (77.2)	160 (90.9)
History of receiving COVID-19 vaccine		
Yes	0 (0)	79 (44.9)
No	171 (100)	97 (55.1)
Having relative(s) who had already received COVID-19 vaccine		
Yes	100 (58.5)	110 (62.5)
No	71 (41.5)	66 (37.5)
Having relative(s) with COVID-19 infection		
Yes	9 (5.3)	16 (9.1)
No	162 (94.7)	160 (90.9)

Values are reported as the median (IQR) or n (%)

*ANC* antenatal care, *COVID-19* coronavirus disease 2019, *IQR* interquartile range

^a^Each participant might have had more than one disease

^b^Including cancer and chronic lung, kidney, cardiovascular and cerebrovascular diseases

### Levels of worry about COVID-19 infection

Table 2 shows the levels of worry about COVID-19 infection among pregnant women and their spouses. The rate of responses that were defined as ‘worry’ was 86.6% (95% CI 81.4%, 91.7%) among the pregnant women, which was significantly higher than the 73.9% rate (95% CI, 67.3%, 80.4%) observed among the husbands (p = 0.003). However, no characteristic of the couples was found to be significantly related to their worry.

**Table 2:** Level of worry about COVID-19 infection and COVID-19 vaccine confidence and acceptance among pregnant women and spouses

Characteristic		Pregnant women	Spouses
		(n = 171)	(n = 176)
Level of worry about COVID-19 infection			
Worry	Very worried	75 (43.9)	54 (30.7)
	Somewhat worried	73 (42.7)	76 (43.2)
No worry	Not very worried	18 (10.5)	35 (19.9)
	Not worried at all	5 (2.9)	11 (6.2)
Confidence in the safety of vaccination during pregnancy			
Yes	Strongly agree	14 (8.2)	16 (9.1)
	Agree	56 (32.7)	66 (37.5)
No	Neutral	36 (21.1)	31 (17.6)
	Disagree	46 (26.9)	41 (23.3)
	Strongly disagree	19 (11.1)	22 (12.5)
Confidence in the efficacy of vaccination during pregnancy			
Yes	Strongly agree	12 (7.0)	19 (10.8)
	Agree	61 (35.7)	61 (34.7)
No	Neutral	35 (20.5)	37 (21.0)
	Disagree	49 (28.6)	40 (22.7)
	Strongly disagree	14 (8.2)	19 (10.8)
Acceptance of vaccination during pregnancy			
Yes	Strongly agree	29 (16.9)	34 (19.3)
	Agree	75 (43.9)	74 (42.1)
No	Neutral	27 (15.8)	22 (12.5)
	Disagree	33 (19.3)	39 (22.1)
	Strongly disagree	7 (4.1)	7 (4.0)
Accepted gestational age for vaccination^a^ (weeks)			
< 14 (first trimester)		12 (11.5)	10 (9.3)
14–28 (second trimester)		58 (55.8)	65 (60.2)
> 28 (third trimester)		40 (38.5)	43 (39.8)

Values are reported as n (%)

*COVID-19* coronavirus disease 2019

^a^Determined only in 104 women and 108 husbands who accepted vaccination during pregnancy. Each participant might accept more than one trimester of pregnancy

### Accepting attitude toward COVID-19 vaccination during pregnancy

The most accepted vaccine by the pregnant women was the messenger ribonucleic acid (mRNA) vaccine (71.9%), followed by the adenovirus vector vaccine (48.0%) and inactivated viral vaccine (31.6%); conversely, the most accepted vaccine by the husbands was the adenovirus vector vaccine (67.6%), followed by the mRNA vaccine (65.3%) and inactivated viral vaccine (49.4%).

Approximately 40.9% (95% CI 33.5%, 48.4%) of pregnant women and 46.6% (95% CI 39.2%, 54.0%) of husbands were confident in the safety of COVID-19 vaccination during pregnancy. Similarly, the rates of confidence in vaccine efficacy were less than 50% among pregnant women and husbands: 42.7% (95% CI 35.2%, 50.2%) and 45.5% (95% 38.0%, 52.9%), respectively (Table 2).

The rates of an accepting attitude toward COVID-19 vaccination during pregnancy were 60.8% (95% CI 53.4%, 68.2%) among pregnant women and 61.4% (95% CI 54.1%, 68.6%) among the male partners (Table 2). More than half (58/104, 55.8%) of pregnant women who accepted vaccination preferred to be vaccinated in the second trimester. In the same direction, most (65/108, 60.2%) husbands who had an accepting attitude favored their wives being vaccinated in the second trimester.

The three most common reasons for declining COVID-19 vaccination during pregnancy among the pregnant women were fear of harm to the baby (58.2%), fear of side effects (17.9%) and mistrust of vaccine efficacy (11.9%). The top three reasons for husbands’ unwillingness to have their wives vaccinated for COVID-19 were fear of harm to the baby (75.0%), mistrust of vaccine efficacy (11.7%) and fear that their wives might have adverse effects (8.8%).

### Factors associated with COVID-19 vaccine acceptance

Univariate analysis demonstrated that worry about COVID-19 infection, confidence in vaccine safety, confidence in vaccine efficacy, and having a husband who favored COVID-19 vaccination for his wife during pregnancy were significantly associated with the acceptance of COVID-19 vaccination during pregnancy among pregnant women (Table 3). However, only the latter was identified as an independent predictor of COVID-19 vaccine acceptance after being incorporated into a multivariate logistic regression model (Table 4). The adjusted OR was 4.82 (95% CI 2.34, 9.94).

**Table 3:** Univariate analysis of factors associated with acceptance of COVID-19 vaccination during pregnancy among pregnant women

Characteristic	Vaccine acceptance		Crude OR
	Yes	No	(95% CI)
	(n = 104)	(n = 67)	
Age (years)^a^	28 (24–33)	29 (23–34)	
Prepregnancy body mass index (kg/m^2^)			
< 18.5	16 (15.4)	9 (13.4)	Reference
18.5–24.9	52 (50.0)	30 (44.8)	0.98 (0.38, 2.48)
25.0–29.9	23 (22.1)	14 (20.9)	0.92 (0.32, 2.65)
≥ 30.0	13 (12.5)	14 (20.9)	0.52 (0.17, 1.59)
Gestational age (weeks)^a^	25 (18–31)	28 (18–31)	
Number of ANC visits			
1–3	30 (28.9)	21 (31.3)	Reference
4–7	54 (51.9)	34 (50.8)	1.11 (0.55, 2.25)
≥ 8	20 (19.2)	12 (17.9)	1.17 (0.47, 2.89)
Number of children			
0	49 (47.1)	29 (43.3)	Reference
1	44 (42.3)	23 (34.3)	1.13 (0.57, 2.24)
≥ 2	11 (10.6)	15 (22.4)	0.43 (0.18, 1.07)
Education (years)			
Primary education	8 (7.7)	6 (8.9)	Reference
Secondary education	58 (55.8)	39 (58.2)	1.12 (0.36, 3.47)
College	11 (10.6)	5 (7.5)	1.65 (0.37, 7.37)
Bachelor’s degree or higher	27 (25.9)	17 (25.4)	1.19 (0.35, 4.04)
Occupation			
Public officer	14 (13.5)	4 (6.0)	2.68 (0.79, 9.09)
Business owner	10 (9.6)	7 (10.4)	1.09 (0.37, 3.26)
Employee	46 (44.2)	30 (44.8)	1.17 (0.59, 2.33)
Unemployment	34 (32.7)	26 (38.8)	Reference
Monthly income			
< $500	63 (60.6)	43 (64.2)	Reference
$500–$999	36 (34.6)	22 (32.8)	1.12 (0.58, 2.15)
≥ $1000	5 (4.8)	2 (3.0)	1.71 (0.32, 9.20)
Public health insurance coverage			
Yes	96 (92.3)	62 (92.5)	0.97 (0.30, 3.09)
No	8 (7.7)	5 (7.5)	Reference
Any underlying disease			
Yes	22 (21.2)	21 (31.3)	0.59 (0.29, 1.18)
No	82 (78.8)	46 (68.7)	Reference
History of receiving a seasonal flu this year			
Yes	27 (26.0)	12 (17.9)	1.61 (0.75, 3.45)
No	77 (74.0)	55 (82.1)	Reference
Having relative(s) who had already received COVID-19 vaccine			
Yes	61 (58.7)	39 (58.2)	1.02 (0.55, 1.90)
No	43 (41.3)	28 (41.8)	Reference
Having relative(s) with COVID-19 infection			
Yes	8 (7.7)	1 (1.5)	5.50 (0.67, 45.02)
No	96 (92.3)	66 (98.5)	Reference
Worry about COVID-19 infection			
Yes	95 (91.3)	53 (79.1)	2.79 (1.13, 6.87)
No	9 (8.7)	14 (20.9)	Reference
Confidence in vaccine safety			
Yes	56 (53.8)	14 (20.9)	4.42 (2.19, 8.93)
No	48 (46.2)	53 (79.1)	Reference
Confidence in vaccine efficacy			
Yes	59 (56.7)	14 (20.9)	4.96 (2.45, 10.05)
No	45 (43.3)	53 (79.1)	Reference
Having a husband who had already received COVID-19 vaccine			
Yes	49 (47.1)	27 (40.3)	1.32 (0.71, 2.46)
No	55 (52.9)	40 (59.7)	Reference
Having a husband who favored his wife to be vaccinated for COVID-19 during pregnancy			
Yes	81 (77.9)	23 (34.3)	6.74 (3.40, 13.36)
No	23 (22.1)	44 (65.7)	Reference

Values are reported as the median (IQR) or n (%)

*ANC* antenatal care, *CI* confidence interval, *COVID-19* coronavirus disease 2019, *OR* odds ratio

^a^P > 0.05 between pregnant women with and without vaccine acceptance

**Table 4:** Multivariate analysis of factors associated with acceptance of COVID-19 vaccination during pregnancy among pregnant women

Characteristic	Adjusted OR^a^
	(95% CI)
Worry about COVID-19 infection	
Yes	1.55 (0.55, 4.40)
No	1.00
Confidence in vaccine safety	
Yes	1.66 (0.35, 7.97)
No	1.00
Confidence in vaccine efficacy	
Yes	1.85 (0.38, 9.11)
No	1.00
Having a husband who favored his wife to be vaccinated for COVID-19 during pregnancy	
Yes	4.82 (2.34, 9.94)
No	1.00

*CI* confidence interval, *COVID-19* coronavirus disease 2019, *OR* odds ratio

^a^Adjusted for the other variables in the table

On the other hand, univariate analysis revealed that a history of receiving the COVID-19 vaccine, confidence in vaccine safety, and confidence in vaccine efficacy were significant factors for the willingness of husbands to have their wives vaccinated for COVID-19 during pregnancy (Table 5). When the multivariate analysis was performed, only confidence in vaccine safety was identified as an independent predictive factor (adjusted OR 12.56; 95% CI 2.35, 67.18) (Table 6).

**Table 5:** Univariate analysis of factors associated with acceptance of COVID-19 vaccination during pregnancy among male partners

Characteristic	Vaccine acceptance		Crude OR
	Yes	No	(95% CI)
	(n = 108)	(n = 68)	
Age (years)^a^	31 (26–35)	29 (24–34)	
Number of children			
0	32 (47.1)	51 (47.2)	Reference
1	26 (38.2)	41 (38.0)	0.99 (0.51, 1.92)
≥ 2	10 (14.7)	16 (14.8)	1.00 (0.41, 2.48)
Education (years)			
Primary education	7 (6.5)	6 (8.8)	Reference
Secondary education	60 (55.6)	45 (66.2)	1.14 (0.36, 3.63)
College	12 (11.1)	5 (7.4)	2.06 (0.46, 9.30)
Bachelor’s degree or higher	29 (26.8)	12 (17.6)	2.07 (0.58, 7.46)
Occupation			
Public officer	24 (22.2)	8 (11.8)	6.00 (0.92, 39.19)
Business owner	10 (9.3)	12 (17.6)	1.67 (0.25, 11.07)
Employee	72 (66.7)	44 (64.7)	3.27 (0.58, 18.62)
Unemployment	2 (1.8)	4 (5.9)	Reference
Monthly income			
< $500	35 (32.4)	30 (44.1)	Reference
$500–$999	61 (56.5)	33 (48.5)	1.59 (0.83, 3.02)
≥ $1000	12 (11.1)	5 (7.4)	2.06 (0.65, 6.51)
Public health insurance coverage			
Yes	44 (40.7)	20 (29.4)	1.65 (0.86, 3.15)
No	64 (59.3)	48 (70.6)	Reference
Any underlying disease			
Yes	10 (9.3)	3 (4.4)	2.21 (0.59, 8.34)
No	98 (90.7)	65 (95.6)	Reference
History of receiving a seasonal flu this year			
Yes	13 (12.0)	3 (4.4)	2.97 (0.81, 10.82)
No	95 (88.0)	65 (95.6)	Reference
History of receiving COVID-19 vaccine			
Yes	57 (52.8)	22 (32.4)	2.34 (1.24, 4.40)
No	51 (47.2)	46 (67.6)	Reference
Having relative(s) who had already received COVID-19 vaccine			
Yes	72 (66.7)	38 (55.9)	1.58 (0.85, 2.95)
No	36 (33.3)	30 (44.1)	Reference
Having relative(s) with COVID-19 infection			
Yes	11(10.2)	5 (7.4)	1.43 (0.47, 4.31)
No	97 (89.8)	63 (92.6)	Reference
Worry about COVID-19 infection			
Yes	85 (78.7)	45 (66.2)	1.89 (0.96, 3.73)
No	23 (21.3)	23 (33.8)	Reference
Confidence in vaccine safety			
Yes	77 (71.3)	5 (7.4)	31.30 (11.50, 85.20)
No	31 (28.7)	63 (92.6)	Reference
Confidence in vaccine efficacy			
Yes	74 (68.5)	6 (8.8)	22.49 (8.86, 57.07)
No	34 (31.5)	62 (91.2)	Reference

Values are reported as the median (IQR) or n (%)

*CI* confidence interval, *COVID-19* coronavirus disease 2019, *OR* odds ratio

^a^P > 0.05 between husbands with and without vaccine acceptance

**Table 6:** Multivariate analysis of factors associated with acceptance of COVID-19 vaccination during pregnancy among male partners

Characteristic	Adjusted OR^a^
	(95% CI)
History of receiving COVID-19 vaccine	
Yes	1.81 (0.81, 4.02)
No	1.00
Confidence in vaccine safety	
Yes	12.56 (2.35, 67.18)
No	1.00
Confidence in vaccine efficacy	
Yes	2.70 (0.53, 13.81)
No	1.00

*CI* confidence interval, *COVID-19* coronavirus disease 2019, *OR* odds ratio

^a^Adjusted for the other variables in the table

### Actual rate of vaccination during pregnancy among the enrolled women

The majority (n = 100, 96.2%) of the 104 women who accepted vaccination were vaccinated against COVID-19 during pregnancy. Four women who had an accepting attitude toward COVID-19 vaccination were not vaccinated because they delivered before the appointment time for vaccination. Nevertheless, all of them received vaccines in the postpartum period. On the other hand, 51 (76.1%) of the 67 women who were initially reluctant to be vaccinated ultimately received COVID-19 vaccines during pregnancy. The actual rate of being vaccinated among the enrolled women was 88.3% (95% CI 83.0%, 93.0%).

## Discussion

At the time of the study, the highly transmissible Delta variant caused a new surge in COVID-19 infection in Thailand. During that time, four COVID-19 vaccines that were approved by the World Health Organization for emergency use [4], including two inactivated viral vaccines (CoronaVac and BBIBP-CorV), one adenovirus vector vaccine (ChAdOx1 nCoV-19), and one mRNA vaccine (BNT162b2), were available in the country but with a limited supply. These four vaccines, predominantly CoronaVac and ChAdOx1, were offered free of charge to the Thai adult population. Among pregnant persons, the main vaccine used was ChAdOx1 nCoV-19.

Despite national efforts to promote vaccination against COVID-19, the rate of willingness to be vaccinated for COVID-19 during pregnancy was only approximately 60% among Thai pregnant women. This figure was lower than the country’s target of vaccinating 70% of its population to reach herd immunity. In comparison to other middle-income Asian countries, our observed acceptance rate was lower than those found in Indian (82%), Chinese (77%), and Filipino (65%) pregnant women [9, 12], but it was comparable to the 60% rate observed in a Vietnamese pregnant cohort [13]. In line with the finding in wives, the rate of an accepting attitude toward COVID-19 vaccination during pregnancy among Thai husbands was modest. The main reason for the low acceptance rates in the Thai couples was the skeptical view about vaccine safety, as the most common reasons for declining vaccination among our respondents were fear of harm to the baby and fear of vaccine-related adverse effects. Another important reason was mistrust in vaccine efficacy. Our results showed that less than 50% of the couples reported that they were confident in the efficacy of COVID-19 vaccination. These reasons, which are consistent with the results of previous studies [9, 11, 13], suggested that the public fear of new COVID-19 vaccines was a significant barrier to effective immunization. In Thailand, the government used the vaccine prioritization policy and a public relations campaign to promote vaccination among pregnant persons. However, this might only partially improve vaccine uptake because there are many other factors that affect public trust in vaccination, including trust/distrust in the government, infodemic on social media, and anti-vaccination behavior. Other obstacles to policy implementation included the slow onset of vaccine procurement, particularly mRNA vaccines, and a limited supply of vaccines.

We found no association of socioeconomic status, including education, occupation, monthly income and public health insurance coverage, with COVID-19 vaccine acceptance among Thai couples. This might be because pregnant women and husbands perceived that it was the responsibility of the government to provide free vaccination to all Thai citizens. Focusing on results in pregnant women, we observed that the number of ANC visits had no effect on an accepting attitude toward COVID-19 vaccination. These results underscored that healthcare providers’ advice or counseling during ANC visits alone might not provide sufficient motivation for pregnant women to accept COVID-19 vaccines. Furthermore, an observation of no significant impact of having a husband who had already been vaccinated for COVID-19 on the increased rate of COVID-19 vaccine acceptance among pregnant women could be explained by the fact that some husbands experienced vaccine side effects, thereby raising their wives’ concerns about vaccine safety. Nevertheless, our multivariate analysis indicated that having a husband who favored his wife being vaccinated against COVID-19 was associated with a 4.8-fold increase in COVID-19 vaccine acceptance among pregnant women. This implied that husbands’ acceptance of COVID-19 vaccines, which was influenced by confidence in vaccine safety (based on multivariate analysis), played a positive role in their wives’ acceptance of COVID-19 vaccination during pregnancy. In many low- and middle-income Asian countries, including Thailand, where husbands are often the breadwinners and play a crucial role in decision-making for receiving specific treatment or interventions during the antenatal period of their pregnant wives [14–16], involving male partners in the vaccination counseling process should therefore be considered. This may help to enhance the vaccine acceptance rate in pregnant cohorts.

We found that most of the couples considered the second trimester of pregnancy to be the most suitable time for being vaccinated. The potential explanation was that all major organs had already been formed by that time. Hence, they perceived that vaccines were less likely to cause fetal anomalies. In addition, the women would have enough time to be fully vaccinated before delivery, especially those receiving the ChAdOx1 vaccine course, which involves a 2-dose series that requires a long duration of 12 weeks to complete. This information might be useful for physicians in educating couples, planning for the proper gestational age for vaccination and choosing a vaccine that requires a short duration to complete the course.

Our results, which showed that almost all pregnant women who accepted vaccination were finally vaccinated against COVID-19 during pregnancy, indicated that once the women trusted the vaccines, they did not hesitate to be vaccinated if the vaccines were offered to them. In contrast, our observation that approximately 76% of women who were initially unwilling to be vaccinated finally received the COVID-19 vaccine during pregnancy suggested that the attitude of vaccine hesitancy was dynamic and might change over the course of time. The reasons for the change from vaccine refusal to vaccination in our pregnant cohort were multifactorial. These included an upsurge in new SARS-CoV-2 infections and deaths, especially in vulnerable populations, including pregnant women, caused by the Delta strain in the country; the COVID-19 vaccine education campaigns provided by the government; and no report on significant adverse events after being vaccinated for COVID-19 among other pregnant women by the media.

Unlike previous cross-sectional surveys, our longitudinal study was the first to explore the rate of willingness to be vaccinated for COVID-19 and the actual rate of being vaccinated during pregnancy in the same set of pregnant women. Another strength was that our study was conducted on site at the ANC clinic. Therefore, study participants who did not understand any specific question could ask for clarification. In addition, the onsite survey included participants who had various sociodemographic characteristics. This was better than online or web surveys, which tend to include only people who have computer or internet access or younger demographics who are online much of the time and have the ability to participate in the survey. Finally, our study also investigated responses among the husbands of pregnant women. To our knowledge, these have not been studied before.

Given that this study was conducted in Bangkok, the capital city of Thailand, where the daily confirmed cases and deaths from COVID-19 as well as the number of vaccines distributed were highest compared to other regions of the country, the findings of this study might not represent the attitudes of pregnant women and their spouses from other parts of the country. Second, the sample size was calculated based on the primary study outcome. Therefore, the study might be underpowered to detect differences in the secondary outcomes between groups. Third, we did not evaluate some factors, for example, trust/distrust in the government and information/misinformation on social media, which might affect accepting attitudes toward COVID-19 vaccination among the couples. Thus, caution is needed when extrapolating our findings to other groups of pregnant women and spouses.

## Conclusion

Although the rates of accepting attitudes toward COVID-19 vaccination during pregnancy among Thai pregnant women and their spouses were modest, the actual rate of being vaccinated during pregnancy among the women was high. Physicians, hospital administrators, and policymakers should focus on those who show vaccine hesitancy or refusal and implement intensive interventions because there is a possibility to change their attitudes if they have more knowledge and gain more trust in the vaccines. Our findings also serve as basic information that can be applied to the development of future vaccination campaigns against emerging infectious diseases among pregnant women.

## Data Availability

The data used in the current study are available from the corresponding author upon reasonable request.

## Abbreviations

ANC: Antenatal care
CI: Confidence interval
COVID-19: Coronavirus disease 2019
IQR: Interquartile range
OR: Odds ratio
SARS-CoV-2: Severe acute respiratory syndrome coronavirus 2
